# Using BTM to reconstruct a complex dorsal hand wound with segmental loss of EDC tendon: Case report and review of the literature

**DOI:** 10.1016/j.jpra.2023.10.013

**Published:** 2023-10-30

**Authors:** Thomas Whitton, Nanda Kandamany

**Affiliations:** a Department of Plastic and Reconstructive Surgery, Royal Hobart Hospital, Tasmania, Australia; b School of Medicine, University of Tasmania, Hobart, Australia

**Keywords:** Hand, Reconstruction, Extensor tendon, BTM

## Abstract

We present the case of a 65 year old man who sustained a complex dorsal hand degloving injury with segmental loss of EDC tendon to middle finger, which was reconstructed using BTM. He returned to near full function despite not having a tendon reconstruction, and the uninjured tendons were able to glide without restriction beneath the BTM. We review the case and the literature surrounding the use of BTM in this clinical scenario.

## Case

**Figure 1:**
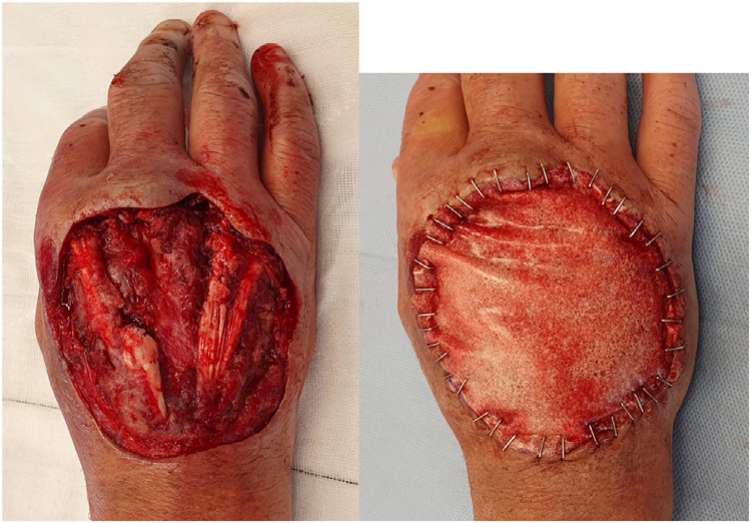
Right hand dorsum wound following debridement - demonstrating soft tissue defect, segmental loss of EDC tendon to middle finger and partial injury to EDC tendon to ring finger and their paratenon (left image). Following application of BTM (right image).

We present the case of a 65-year-old right hand dominant man who was admitted to hospital following a mountain-bike accident, from which he sustained a severe closed head injury and a large degloving injury to the dorsum of his right hand. The wound was contaminated with gravel and organic debris, with a 3 cm segmental loss of the extensor digitorum communis (EDC) tendon to middle finger in zone VI and its juncturae tendinum, and a partial injury to EDC tendon to ring finger (approx. 50 %). Our patient was previously well and a non-smoker. Initial wound debridement was undertaken in the operating theatre with contaminated and non-viable tissue debrided and a negative pressure wound therapy (NPWT) dressing applied (V.A.C. KCI, San Antonio, Texas, USA) (Figure 1).

**Figure 2:**
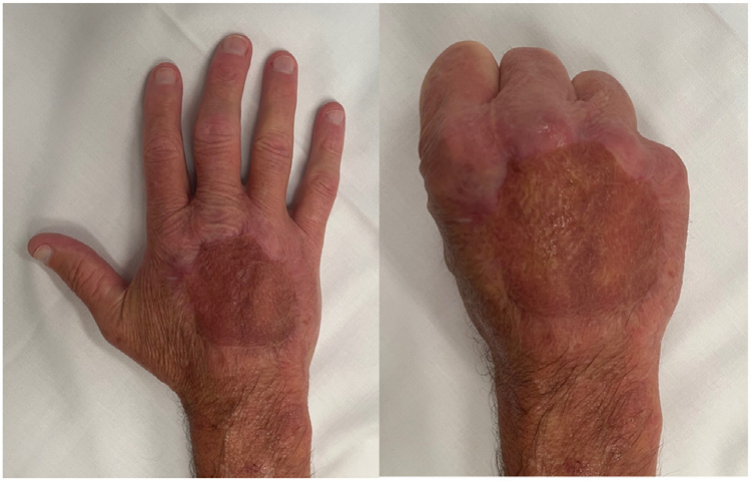
Appearance of hand at 2-years post-surgery.

An ideal reconstructive plan for this non-graftable wound would utilise a thin and pliable free flap to cover the reconstructed EDC tendon. Unfortunately, our patient's neurological status at the time prohibited him from undergoing a long anaesthetic, and there were concerns regarding his ability to comply with splinting and rehabilitation protocols while recovering from a severe traumatic brain injury. Thus, a decision was made to apply Biodegradable Temporizing Matrix (BTM) (PolyNovo Biomaterials Pty Ltd., Melbourne, Australia) to the wound, which was secured with surgical staples and a NPWT dressing (Figure 2).

The BTM was sufficiently integrated at 4-weeks post application and was delaminated, and a thin split-thickness skin graft taken at 7/1000ths of an inch was applied. The skin graft was successful, showing 100% take at one-week post-surgery. Active range of motion exercises were then commenced under the guidance of a hand physiotherapist.

**Figure 3:**
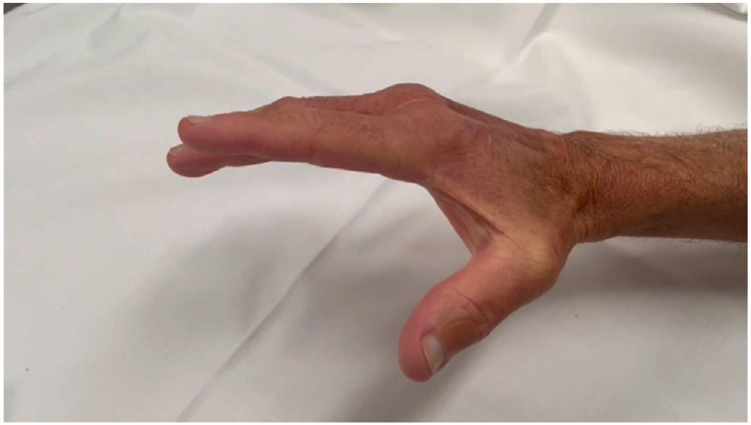
Video demonstrating active range of motion.

At review three-months post-surgery, our patient had made remarkable progress. He could make a fist with unrestricted range of motion in finger flexion. More surprisingly, he had active extension of the middle finger, with only approximately 30° of extensor lag despite no tendon reconstruction having been undertaken. On further review 15-months post-surgery, our patient had made further improvements with only 10° of extensor lag present in the middle finger. Power in resisted finger extension was equivalent to that on the contralateral hand. The neodermis and skin graft were supple and showed no signs of adhesion to the underlying extensor tendons (Figure 3). Our patient was satisfied with both the cosmetic and functional result and reported being able to perform tasks such as gripping, writing, buttoning a shirt and gardening without restriction

Ultrasound scan performed two-years post-operatively demonstrated a persisting tendon gap of approximately 35 mm in the EDC to middle finger, with both proximal and distal ends gliding normally, pulling through tissue that had formed beneath the BTM neodermis. The other extensor tendons appeared to glide smoothly without adhesion beneath the BTM neodermis.

## Discussion

BTM is a synthetic dermal substitute that is used to reconstruct a variety of complex wounds including burns, necrotising fasciitis and traumatic wounds, including those on the upper and lower extremities.^1^, ^2^, ^3^, ^4^ In this case, BTM quickly integrated into the traumatic wound bed forming a stable neodermis within four weeks. In doing so, BTM converted a non-graftable wound bed that would previously have required free flap reconstruction into a healthy vascularised wound bed amenable to skin grafting.^1^^,^^3^ Previous authors have reported on the ability for tendons to glide beneath the BTM neodermis.^5^ To the authors’ knowledge, no report exists in the literature describing the spontaneous return of function in an unrepaired/unreconstructed tendon with a segmental loss following the application of BTM and propose that this is due to formation of scar or pseudotendon beneath the BTM neodermis. These findings further support the evidence that BTM is safe to apply directly over tendons without concern for the formation of adhesions or reduction in tendon glide and hand function.

## Funding

Neither author received funding or sponsorship.

## Declaration of Competing Interest

None.

## Appendix Supplementary materials

Image, video 1
